# Supplementary material for: Visual and Ultrasensitive Detection of a Coronavirus Using a Gold Nanorod Probe under Dark Field
Source: Biosensors (Basel). 2022 Dec 8;12(12):1146. doi: 10.3390/bios12121146 (PMC9775988; doi:10.3390/bios12121146)
Supplement: Supplementary file 1 [file biosensors-12-01146-s001.zip › biosensors-2039381-supplementary.pdf]

biosensors

MDPI

Supporting Information

Article

Visual and Ultrasensitive Detection of a Coronavirus Using a Gold Nanorod Probe under Dark Field

Xuejia Qian [1,2,3,†], Yuanzhao Shen [1,†], Jiasheng Yuan [1], Chih-Tsung Yang [4,*] and Xin Zhou [1,2,3,*]

[1] College of Veterinary Medicine, Institute of Comparative Medicine, Yangzhou University, Yangzhou 225009, China
[2] Jiangsu Co-Innovation Center for Prevention and Control of Important Animal Infectious Diseases and Zoonoses, Yangzhou University, Yangzhou 225009, China
[3] Joint International Research Laboratory of Agriculture and Agri-Product Safety, The Ministry of Education of China, Yangzhou University, Yangzhou 225009, China
[4] Future Industries Institute, University of South Australia, Mawson Lakes Campas, Adelaide, SW 5095, Australia
* Correspondence: chih-tsung.yang@unisa.edu.au (C.-T.Y.); zhou_xin@126.com (X.Z.)
† These authors contributed equally to this work.

Citation: Qian, X.; Shen, Y.; Yuan, J.; Yang, C.-T.; Zhou, X. Visual and Ultrasensitive Detection of a Coronavirus Using a Gold Nanorod Probe under Dark Field. *Biosensors* **2022**, *12*, 1146. https://doi.org/10.3390/bios12121146

Received: 9 November 2022
Accepted: 6 December 2022
Published: 8 December 2022

Publisher's Note: MDPI stays neutral with regard to jurisdictional claims in published maps and institutional affiliations.

Copyright: © 2022 by the authors. Licensee MDPI, Basel, Switzerland. This article is an open access article distributed under the terms and conditions of the Creative Commons Attribution (CC BY) license (https://creativecommons.org/licenses/by/4.0/).

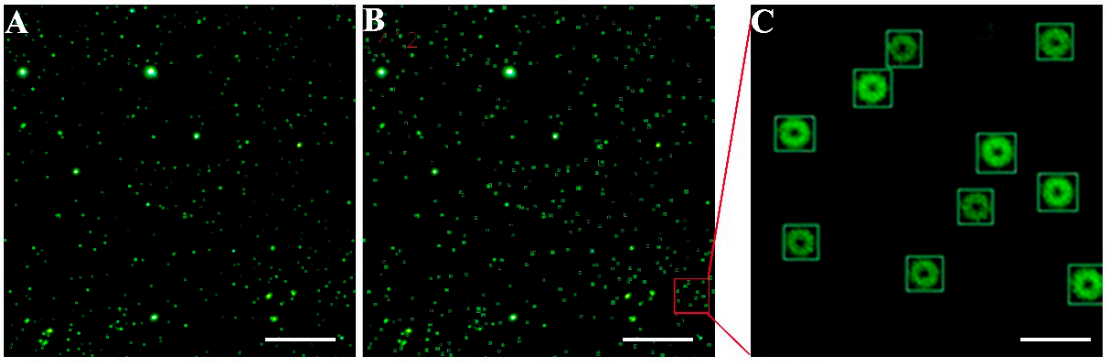

Figure S1. Counting diagram for individual fields of view. (A) A DFM image of the captured PEDVs labeling with GNR probes. (B) The count of green halos formed from GNRs in DFM image by our **Counting software**. The **Counting software** automatically identifies and counts the green halos in dark field images and removes impurities that look similar but are different in color and size from the green halos formed by GNR. (C) The enlargement of the selected area in the red square of (B). Scale bar: 20 μm (A，B) and 2 μm (C).

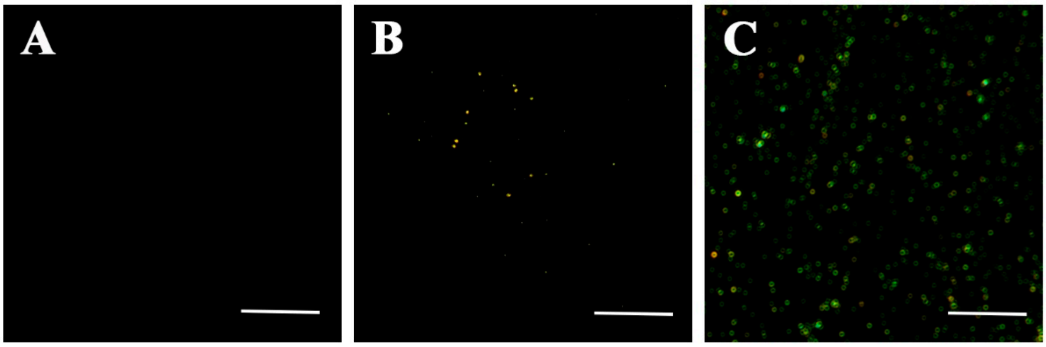

Figure S2. Characterization of GNRs on silicon wafer under the dark field. (A) Pure silicon wafer. (B) Ultrapure water control. (C) Silicon wafer with GNR particles diluted in ultrapure water. Scale bar: 10 μm.

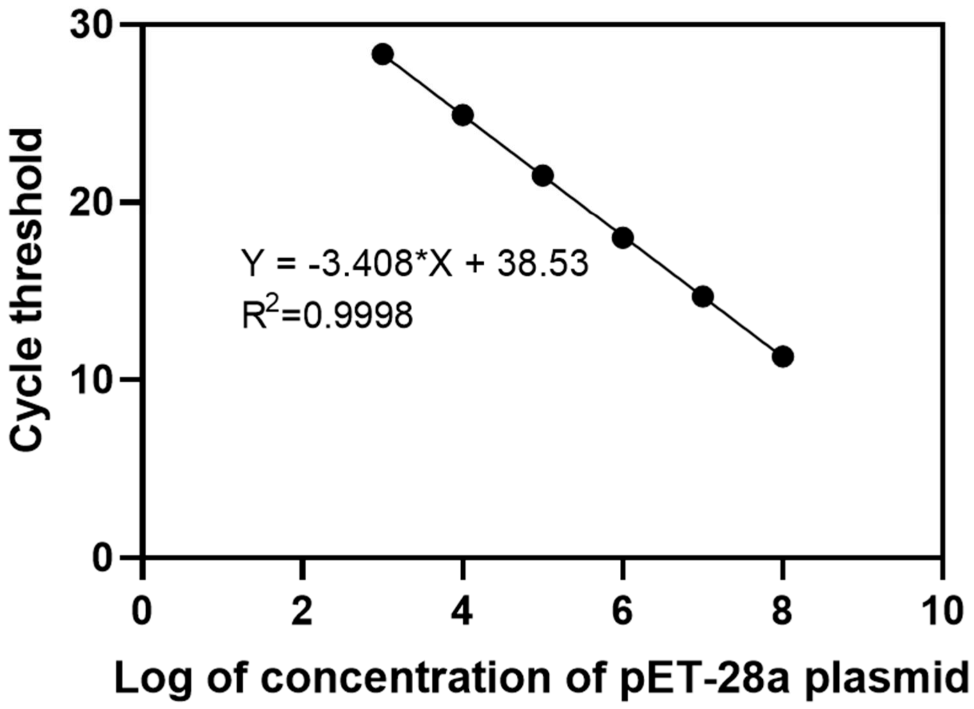

Figure S3. Standard quantification curve of PEDV qPCR assay to detect samples in different biological matrices.

Table S1. The primers for qPCR to amplify 108 bp fragment of PEDV CV777 strain genome (GenBank: AF353511.1).

**The primers for qPCR:**<br>Forward primer: 5'-TGGTGGCTGCTGTCAAGGA-3';<br>Reverse primer: 5'- TGCCGCTGTTGTCAGACTTTT -3'
**The 108 bp fragment sequence:**<br>TGGTGGCTGCTGTCAAGGATGCACTTAAATCTTTGGGTATTGGA-<br>GAAAATCCTGACAGGCATAAGCAACAGCAGAAGCCTAAGCAGGAAAAGTCTGACAACAGCGGCA
